# Establishment and validation of a predictive nomogram for central venous catheter-related thrombosis in cancer patients: a retrospective nested case-control study

**DOI:** 10.3389/fonc.2024.1418273

**Published:** 2024-08-16

**Authors:** Xuexing Wang, Xiao Dai, Yuan He, Jie Chu, Yufeng Wang

**Affiliations:** ^1^ Department of Oncology, Anning First People's Hospital Affiliated to Kunming University of Science and Technology, Kunming, Yunnan, China; ^2^ Cadre Medical Department, The Third Affiliated Hospital of Kunming Medical University, Kunming, Yunnan, China; ^3^ Department of Oncology, Dongxing District People's Hospital, Neijiang, Sichuan, China; ^4^ Department of Oncology, Ziyang Hospital of Sichuan University West China Hospital, Ziyang, Sichuan, China

**Keywords:** nomogram, catheter-related thrombosis, risk factor, logistic regression, nested case-control study

## Abstract

**Background:**

Catheter-related thrombosis (CRT) is a common complication for patients who receive central venous catheter (CVC) placement. This study investigated the risk factors for CRT and developed a nomogram for CRT prediction among cancer patients.

**Methods:**

This nested case-control study was conducted in the Third Affiliated Hospital of Kunming Medical University between January 2019 and February 2021. Univariable and multivariable logistic regression analyses were used to identify the risk factors for CRT. A nomogram was developed to predict CRT. Receiver operating curves (ROC), calibration curves, and decision curves were used to evaluate the performance of the nomogram in the training and validation sets.

**Results:**

A total of 4,691 cancer patients were included in this study. Among them, 355 (7.57%) had CRT, and 70% of CRTs occurred in the first week of insertion. Among the 3,284 patients in the training set, the multivariable analysis showed that nine characteristics were independently associated with CRT, and a nomogram was constructed based on the multivariable analysis. The ROC analysis indicated good discrimination in the training set (area under the curve [AUC] = 0.832, 95% CI: 0.802–0.862) and the testing set (AUC = 0.827, 95% CI: 0.783–0.871) for the CRT nomogram. The calibration curves showed good calibration abilities, and the decision curves indicated the clinical usefulness of the prediction nomograms.

**Conclusion:**

The validated nomogram accurately predicts CRT occurrence in cancer patients. This model may assist clinicians in developing treatment plans for each patient.

## Introduction

According to Global Cancer Statistics 2020, about 19.3 million new cancer cases and 10.0 million cancer deaths occurred during 2020. The estimated cumulative incidence in China was 23.25% and 18.78% in men and women between 0 years and 74 years (1). Central venous catheters (CVCs) facilitate chemotherapy administration, parenteral nutrition, blood product transfusion, and rehydration and are widely used in cancer patients (2). CVC placement can also facilitate the transition of intermittent chemotherapy patients from hospitals to intermediate care settings at home (3, 4). In the USA, approximately eight million CVCs are inserted annually (5).

Although CVC placement offers many advantages to cancer patients, a study reported that over 15% of patients who received a CVC would develop complications (6), including mechanical complications, infectious complications, and thrombotic complications. Among these complications, catheter-related thrombosis (CRT) is frequently observed and has drawn great attention from clinical workers in recent years. Pulmonary embolism caused by CRT is a significant complication that can threaten the lives of cancer patients (7). The reported incidence varies among study populations and periods (8–10). The incidence of symptomatic CRT would vary from 5% to 30.3%, while the incidence of asymptomatic CRT would be higher (11).

Still, few researchers have focused on developing CRT occurrence models in cancer patients. The pathogenesis of CRT is affected by many factors, such as patient characteristics, anticoagulation drug use, catheter size, catheter location, and many other factors (12). Previous studies for risk factors of CRT mainly focused on a group of patients under specific pathological states (13–15) and were performed with less than a thousand patients (16).

Given CRT’s high incidence and harmfulness, it is urgent to understand its risk factors in detail and develop precautions accordingly. Therefore, this study aimed to investigate the risk factors for CRT and develop a nomogram for CRT prediction among cancer patients.

## Methods

### Study design and patients

This nested case-control study was conducted in the Third Affiliated Hospital of Kunming Medical University between January 2019 and February 2021 and included cancer patients who underwent CVC insertion. The inclusion criteria were (1) patients confirmed with tumors by histopathological, cytological, and imaging examinations; (2) inpatients who underwent CVC placement (all CVCs used in this study were nontotal implantable single-lumen 14 G CVCs 20 cm in length) through right internal jugular vein catheterization; and (3) the presence of thrombosis was confirmed by ultrasound examination. The exclusion criteria were (1) patients with a bleeding tendency or history of exposure to anticoagulant drugs (long-term oral anticoagulant drugs or discontinuation of anticoagulant drugs for less than 2 weeks outside the hospital or in the hospital within 2 weeks; (2) patients with prothrombin time—international normalization ratio (PT-INR) greater than 1.3 after warfarin treatment; (3) severe heart, lung, or renal insufficiency, or referral to ICU or CCU during hospitalization; (4) diagnosis of deep vein thrombosis (DVT); or (5) hematological cancers. This study was reviewed and approved by the Institutional Ethics Committee of Yunnan Cancer Hospital (No. KYLX2023–101). The requirement for individual informed consent was waived because of the retrospective nature of the study.

**Figure 1:**
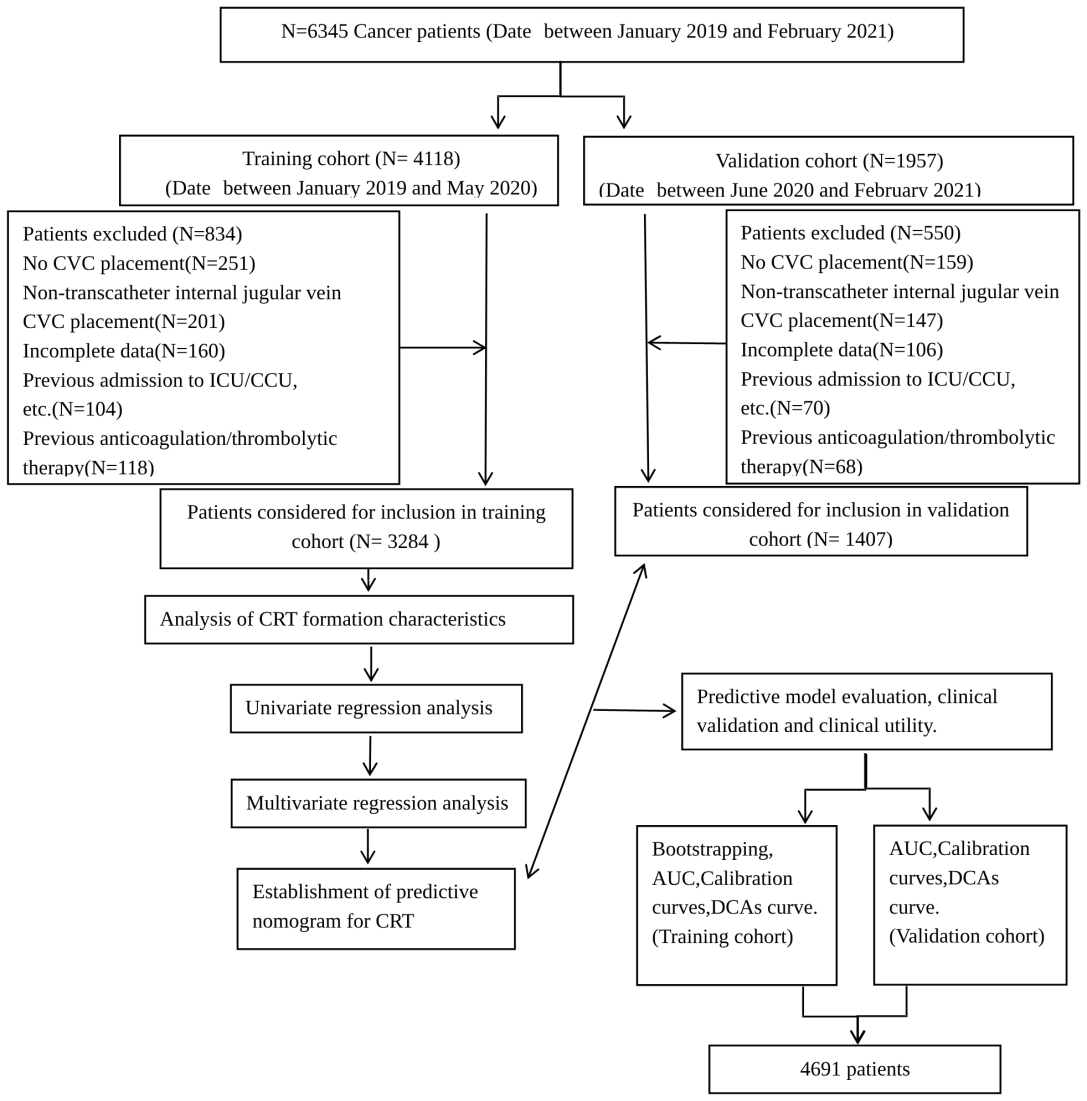
Flowchart of this study. This flow diagram indicates the inclusion and exclusion of patients and the workflow of the method present in this study.

The patients were divided into the training and validation sets according to the admission time: the patients admitted between January 2019 and May 2020 were included in the training set, and those admitted between June 2020 and February 2021 were included in the validation set (**Figure 1**).

### Routine CVC care

The routine CVC protocols in place during the study period included the following: A portable ultrasound was performed before placing CVCs to ensure a suitable vein could be found. CVC nurses performed all the insertions in a separate sterile ward. Chest radiography was routinely performed to identify the position of the catheter tip after CVC insertion. The CVCs were flushed with 10 mL of a 0.9% NaCl solution before and after clinical treatment and during CVC occlusions. In addition to covering the catheter entrance site with dressings every week and thoroughly disinfecting it, the site was cleaned each week. The superior and inferior clavicular, cephalic, femoral, noble, and splenic veins were routinely examined by portable ultrasound during CVC implantation, focusing on the internal jugular vein to detect CVC tip malposition and the presence of thrombosis. Other veins could also be examined as per clinical indications.

### Data collection

CRT was defined as vascular injury caused by various catheter insertions, venous stasis caused by catheter indwelling, catheters in continuous exercise veins, and cancer-related hypercoagulability caused by the formation of blood clots in the outer or inner wall of the catheter (17). The clinical characteristics, medical history, clinical indicators, and biochemical indicators were collected from the medical records.

The clinical indicators included age, gender, diagnosis, staging, Karnofsky Performance Status (KPS) score, height, weight, body mass index (BMI), history of smoking, drinking, blood transfusion, surgery, hypertension, diabetes, hyperlipidemia, CVC complications, thrombosis, and hypercoagulable state. All patients were re-staged according to the seventh AJCC TNM staging manual (10).

The laboratory indicators included blood routine (red blood cells, white blood cells, platelets, hemoglobin), coagulation mechanism (PT, INR, PT ratio, APTT, TT, FIB, plasma antithrombin III, fibrinogen degradation products, and D-dimer). The biochemical indicators included albumin, globulin, direct bilirubin, indirect bilirubin, triglyceride, total cholesterol, and fasting blood glucose. Medical history included hormone usage and infection states during catheterization.

### Statistical analysis

According to the logistic regression prediction model, the sample size of CRT patients should be five to 10 times the number of risk factors used in regression models to avoid excessive prediction error (18). This study included 36 variables as potential risk factors. In order to ensure the accuracy of prediction, the sample size of patients with thrombosis in this study should be at least 180 cases.

SPSS 26.0 (IBM, Armonk, NY, USA) and the R package (version 4.2.0) were used for statistical analysis. The continuous variables were classified into categorical variables according to clinical experience (**Supplementary Table S1**). The categorical variables were expressed as *n* (%) and compared using the Chi-square test. Before further analysis, variables with a missing rate of > 20% were removed, while variables with a missing rate of < 20% were interpolated using multiple interpolation methods. In the training set, univariable and multivariable logistic regression analyses were used to identify the risk factors for CRT. The characteristics with *p <* 0.25 in the univariable logistic regression were included in the multivariable logistic regression. The nomogram was formulated using multivariable logistic regression analysis (**Supplementary Table S1**). Nomogram performance was evaluated using the concordance index (C-index) and assessed by comparing nomogram-predicted and observed probabilities of CRT formation. Bootstrapping with 1,000 resamples was applied to these experiments. A higher C-index indicated a more accurate prognostic stratification. Calibration curves were plotted, and the Hosmer–Lemeshow test (HL test) *p*-value was calculated to evaluate the model’s over-fitting performance (19). The decision curve analysis (DCA) was used to evaluate the clinical utility of nomogram prediction. The ROC analysis and Delong test P value were used to compare the predictive values for the CRT risk nomogram model. Based on the nomogram, all patients were divided into the low-risk group (predicted incidence: 0%–30%), medium-risk group (predicted incidence: 30%–60%), and high-risk group (> 60%) according to the CRT-predicted incidence, and the actual incidence of CRT was compared among the three groups. All statistical tests were two-tailed, and P<0.05 was considered statistically significant.

## Results

### Study population and characteristics

A total of 6,345 consecutive cancer patients received CVC insertion between January 2019 and February 2021, but 1,654 were excluded according to the inclusion and exclusion criteria. The indwelling time was 0.31 (0.10, 1.56) months. According to the admission time, 3,284 and 1,407 patients were included in the training and validation sets, respectively (**Figure 1**). A total of 355 CRT events were observed in the 4,691 patients. The overall CRT incidence was 7.60%, with 262 and 93 patients in the training and validation sets, respectively. The most common CRT occurrence site was cervical vascular, accounting for 32.50%, and the mean time between insertion and thrombosis was 10.01 days ± 6.05 days (**Supplementary Figure S1**). There were no significant differences in age, activity amount, operation history, or hyperlipidemia between the two sets (all *p* > 0.05). However, other characteristics showed significant differences between the two sets (all *p* < 0.05) (**Supplementary Table S2**).

### Characteristics for predicting CRT

In the training set, there were no significant differences in age, gender, type of cancer, history of chemotherapy, BMI, smoking history, drinking history, APTT, or total cholesterol between patients with or without CRT (all *p* > 0.05). However, the TNM stage, number of CVC insertions, activity amount, operation history, KPS, co-infection, hormone use, medical history, and laboratory examination results showed significant differences between the two groups (all *p* < 0.05) (**Table 1**).

**Table 1:** Demographic and clinicopathological characteristics of patients in the training set.

Characteristic	Non-CRT (*n* = 3,022)	CRT (*n* = 262)	*χ*²	*p*-value
Age	53.58 (12.09)	56.17 (12.20)		0.434
Gender			0.3	0.314
Male	1045 (34.6)	95 (36.3)		
Female	1977 (65.4)	167 (63.7)		
TNM stage			45.614	<0.001
I	785 (26.0)	37 (14.1)		
II	723 (23.9)	46 (17.6)		
III	648 (21.4)	55 (21.0)		
IV	866 (28.7)	124 (47.3)		
Type of cancer			8.761	0.555
Genital cancers	1184 (39.2)	105 (40.1)		
Respiratory cancers	777 (25.7)	61 (23.3)		
Digestive cancers	694 (23.0)	57 (21.8)		
Head and neck system tumors	145 (4.8)	13 (5.0)		
Motior system cancers	48 (1.6)	4 (1.5)		
Other cancers*	174 (5.7)	22 (8.3)		
Number of CVC insertions			3.144	0.043
≤2	1254 (41.5)	94 (35.9)		
>2	1768 (58.5)	168 (64.1)		
Activity amount			225.357	<0.001
Hardly	26 (0.9)	37 (14.1)		
Frequently	2996 (99.1)	225 (85.9)		
History of chemotherapy			2.772	0.054
No	614 (20.3)	42 (16.0)		
Yes	2408 (79.9)	220 (84.0)		
Operation history			8.122	0.002
No	1241 (41.1)	84 (32.1)		
Yes	1781 (58.9)	178 (67.9)		
KPS			25.416	<0.001
>80 points	2483 (82.2)	182 (69.5)		
≤80 points	539 (17.8)	80 (30.5)		
BMI (kg/m^2^)				
≤18.4	402 (13.3)	35 (13.4)	2.026	0.567
18.5-23.9	1852 (61.3)	151 (57.6)		
24.0-27.9	615 (20.4)	59 (22.5)		
≥28	153 (5.1)	17 (6.5)		
Smoking history			0.698	0.706
No	2346 (77.6)	198 (75.6)		
Yes	676 (22.4)	64 (24.4)		
Drinking history			1.77	0.413
No	2602 (86.1)	218 (83.2)		
Yes	420 (13.9)	44 (16.8)		
Co-infection			74.871	<0.001
No	2724 (90.1)	190 (72.5)		
Yes	298 (9.9)	72 (27.5)		
Hormone use			34.461	<0.001
No	1470 (48.6)	78 (29.8)		
Yes	1552 (54.1)	184 (70.2)		
History of blood transfusion			20.224	0.002
No	2870 (95.1)	92.4 (237)		
Yes	149 (4.9)	25 (9.5)		
Hypertension			12.502	0.001
No	2634 (87.2)	208 (79.4)		
Yes	388 (12.8)	54 (20.6)		
Diabetes mellitus			5.768	0.016
No	2881 (95.3)	241 (92.0)		
Yes	141 (4.7)	21 (8.0)		
Hyperlipidemia			57.292	<0.001
No	2605 (86.2)	180 (68.7)		
Yes	417 (13.8)	82 (31.3)		
Thrombosis/Hypercoagulability history			594.288	<0.001
No	2965 (98.1)	172 (65.6)		
Yes	57 (1.9)	90 (61.2)		
RBC (10^12^/l)			26.769	<0.001
<3.5	362 (12.0)	60 (22.9)		
3.5-5.5	2622 (86.8)	201 (76.7)		
>5.5	38 (1.3)	1 (0.4)		
WBC (10^9^/l)			23.586	<0.001
<4	593 (19.6)	47 (17.9)		
4-10	1995 (66.0)	148 (56.5)		
>10	434 (14.4)	67 (25.6)		
PLT (10^9^/l)			25.04	<0.001
<100	115 (3.8)	17 (6.5)		
100-300	2140 (70.8)	147 (56.1)		
>300	767 (25.4)	98 (37.4)		
HGB (g/l)			17.869	<0.001
<110	708 (23.4)	92 (35.1)		
≥110	2314 (76.6)	170 (64.9)		
PT (s)			8.474	0.002
≤12.1	776 (25.7)	46 (17.6)		
>12.1	2246 (74.3)	216 (82.4)		
INR			32.227	<0.001
<0.84	77 (2.5)	3 (1.1)		
0.84-1.06	2558 (84.6)	193 (73.7)		
>1.06	387 (12.8)	66 (25.2)		
APTT (s)			0.21	0.358
≤31.3	230 (7.6)	22 (8.4)		
>31.3	2792 (92.4)	240 (91.6)		
TT			3.609	0.045
<14	149 (4.9)	20 (7.6)		
≥14	2873 (95.1)	242 (92.4)		
FIB			19.489	<0.001
<4	1685 (55.8)	109 (41.6)		
≥4	1337 (44.2)	153 (58.4)		
ATIII			42.588	<0.001
≤75	122 (4.0)	34 (4.0)		
>75	2900 (96.0)	228 (87.0)		
FDP			132.326	<0.001
<5	1028 (34.0)	75 (28.6)		
5-10	1792 (59.3)	116 (44.3)		
>10	202 (6.7)	71 (27.1)		
D-Dimer				
0-0.55	529 (17.5)	16 (6.1)	206.965	<0.001
0.56-1.00	329 (10.9)	28 (10.7)		
1.01-2.00	257 (8.5)	47 (17.9)		
2.01-3.00	1699 (56.2)	93 (35.5)		
3.01-4.00	66 (2.2)	22 (8.4)		
>4.00	142 (4.7)	56 (21.4)		
Albumin (g/L)			31.372	<0.001
≤40	745 (24.7)	106 (40.5)		
>40	2277 (75.3)	156 (59.5)		
Albumin/Globulin			15.178	<0.001
≤1.2	351 (11.6)	52 (19.8)		
>1.2	2671 (88.4)	210 (80.2)		
Total bilirubin			8.263	0.004
≤17.1	2766 (91.5)	226 (86.3)		
>17.1	256 (8.5)	36 (13.7)		
Total cholesterol			0.429	0.277
<5.18	1850 (61.2)	155 (59.2)		
≥5.18	1172 (38.8)	107 (40.8)		
Fasting Blood Glucose				
<6.11	2601 (86.1)	213 (81.3)	4.475	0.024
≥6.11	421 (13.9)	49 (18.7)		

Other cancers*: solid tumours other than those listed above, e.g. cancers of the endocrine system: e.g. thyroid cancer, adrenocortical cancer. Tumours of the nervous system: e.g. brain tumours, spinal cord tumours, gliomas, etc.

The results of the univariable logistic regression analysis demonstrated that 26 variables were selected for multivariable logistic regression analysis (**Supplementary Table S3**). The multivariable analysis identified nine characteristics (including TNM stage, activity amount, operation history, co-infection, hormone usage, hyperlipidemia, thrombosis/hypercoagulability history, PLT, and D-Dimer) as being risk factors for CRT (**Table 2**).

**Table 2:** Multivariable logistic regression analysis for CRT in the training set.

Characteristics	OR (95%CI)	*p*-value
TNM stage		
I	Reference	
II	1.454 (0.856-2.468)	0.166
III	1.706 (1.021-2.849)	0.041
IV	2.177 (1.353-3.503)	<0.001
Activity amount		
Hardly	Reference	
Frequently	0.084 (0.043-0.166)	<0.001
Operation history		
No	Reference	
Yes	1.468 (1.058-2.037)	0.022
Co-infection		
No	Reference	
Yes	1.796 (1.219-2.647)	0.003
Hormone use		
No	Reference	
Yes	2.47 (1.755-3.476)	<0.001
Hyperlipidemia		
No	Reference	
Yes	2.448 (1.717-3.493)	<0.001
Thrombosis/Hypercoagulability history		
No		
Yes	20.831 (13.529-32.074)	<0.001
PLT(10^9/l)		
<100	Reference	
100-300	0.783 (0.396-1.551)	0.484
>300	1.318 (0.654-2.657)	0.440
D-Dimer		
0-0.55	Reference	
0.56-1.00	2.687 (1.337-5.398)	0.005
1.01-2.00	4.63 (2.389-8.972)	<0.001
2.01-3.00	1.724 (0.955-3.115)	0.071
3.01-4.00	10.824 (4.763-24.599)	<0.001
>4.00	8.835 (4.466-17.479)	<0.001

### Nomogram for CRT prediction and validation

**Figure 2:**
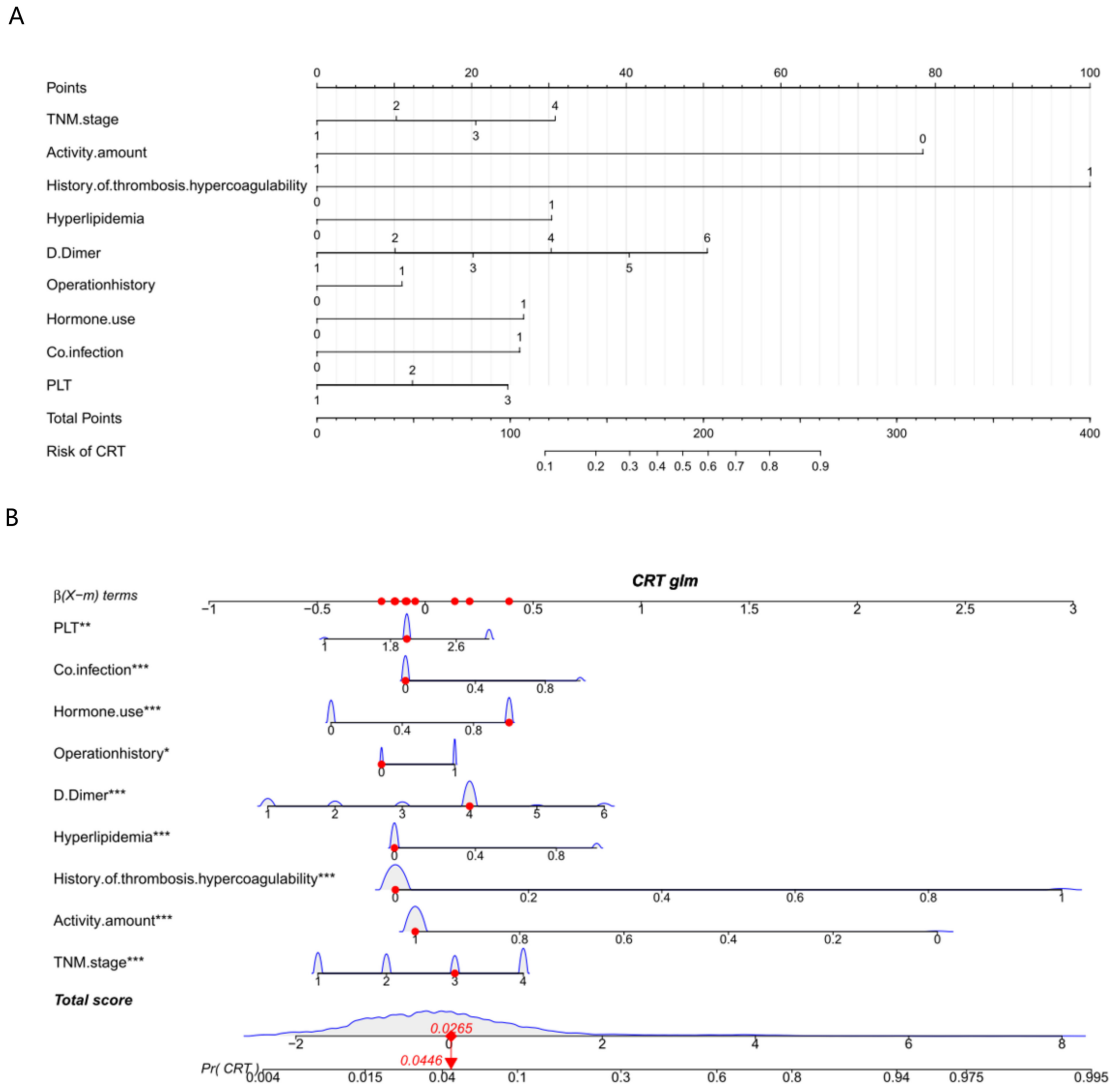
Nomogram **(A)** and interactive nomogram **(B)** for predicting CRT risk in tumor patients after CVC catheterization. There are 10 factors in the CRT prediction nomogram, including TNM stage (stage I = 1, stage II = 2, stage III = 3, stage IV = 4), activity amount (hardly = 0, frequently = 1), operation history (no = 0, yes = 1), co-infection (no = 0, yes = 1), hormone use (no = 0, yes = 1), hyperlipidemia, thrombosis/hypercoagulability history (no = 0, yes = 1), PLT (< 100 = 1, 100–300 = 2, > 300 = 3), and D-dimer (0–0.55 = 1, 0.56–1.00 = 2, 1.01–2.00 = 3, 2.01–3.00 = 4, 3.01–4.00 = 5, > 4.00 = 6). All the points assigned on the top point scale for each factor are summed together to generate a total point score. The total point score is projected on the bottom scale to determine the probability of CRT formation in an individual. As an example, locate the patient’s TNM stage and draw a line straight upward to the “Points” axis to determine the score associated with that TNM stage. Repeat the process for each variable, sum the scores achieved for each covariable, and locate this sum on the “Total points” axis. Draw a line straight down to determine the probability of CRT formation. *P <0.05, **P <0.01, ***P <0.001.

**Figure 3:**
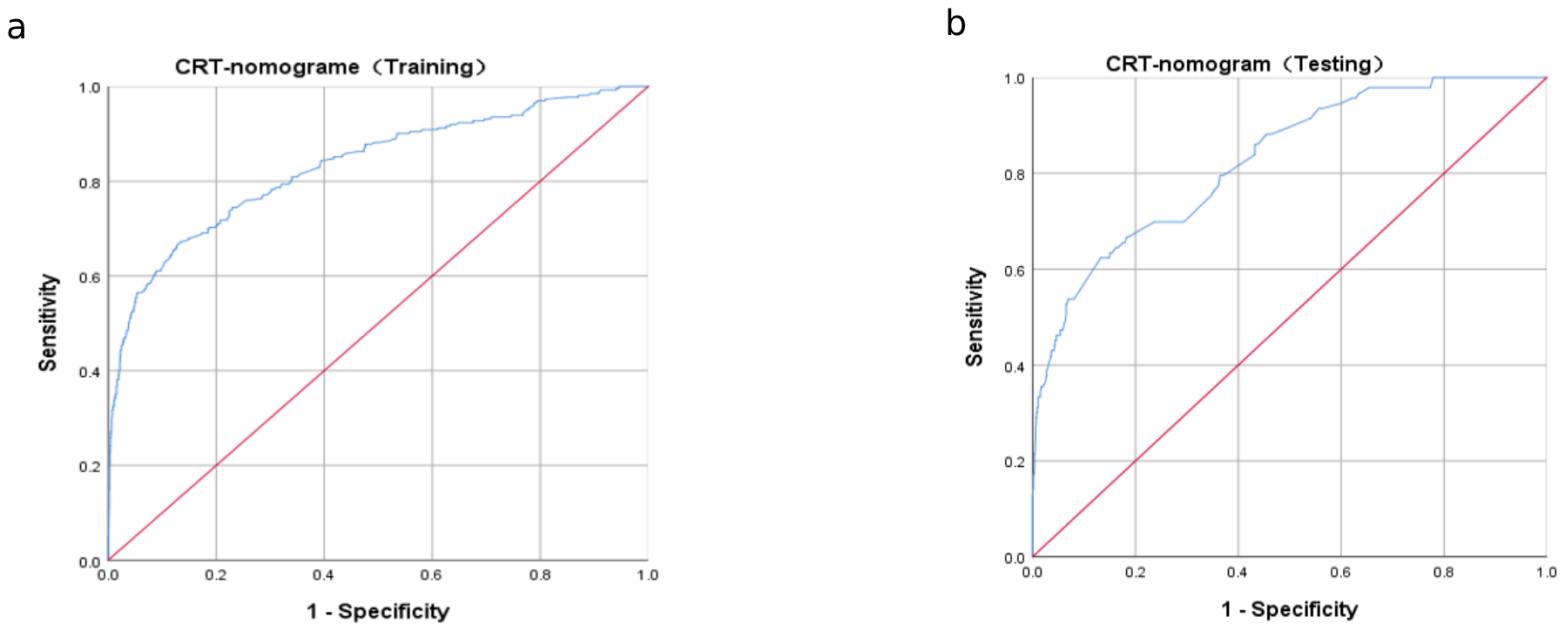
Receiver operating characteristic (ROC) curve analysis for CRT risk prediction. ROC curves of CRT risk prediction in the training set **(A)** and the testing set **(B)**. AUC was calculated using bootstrapping, and its 95% CI was estimated. The *p*-values were two-sided. The AUC and 95% CI in the training set and the testing set were 0.832 (95% CI: 0.802–0.862) and 0.827 (95% CI: 0.783–0.871), respectively, and the Delong test *p* = 0.941.

**Figure 4:**
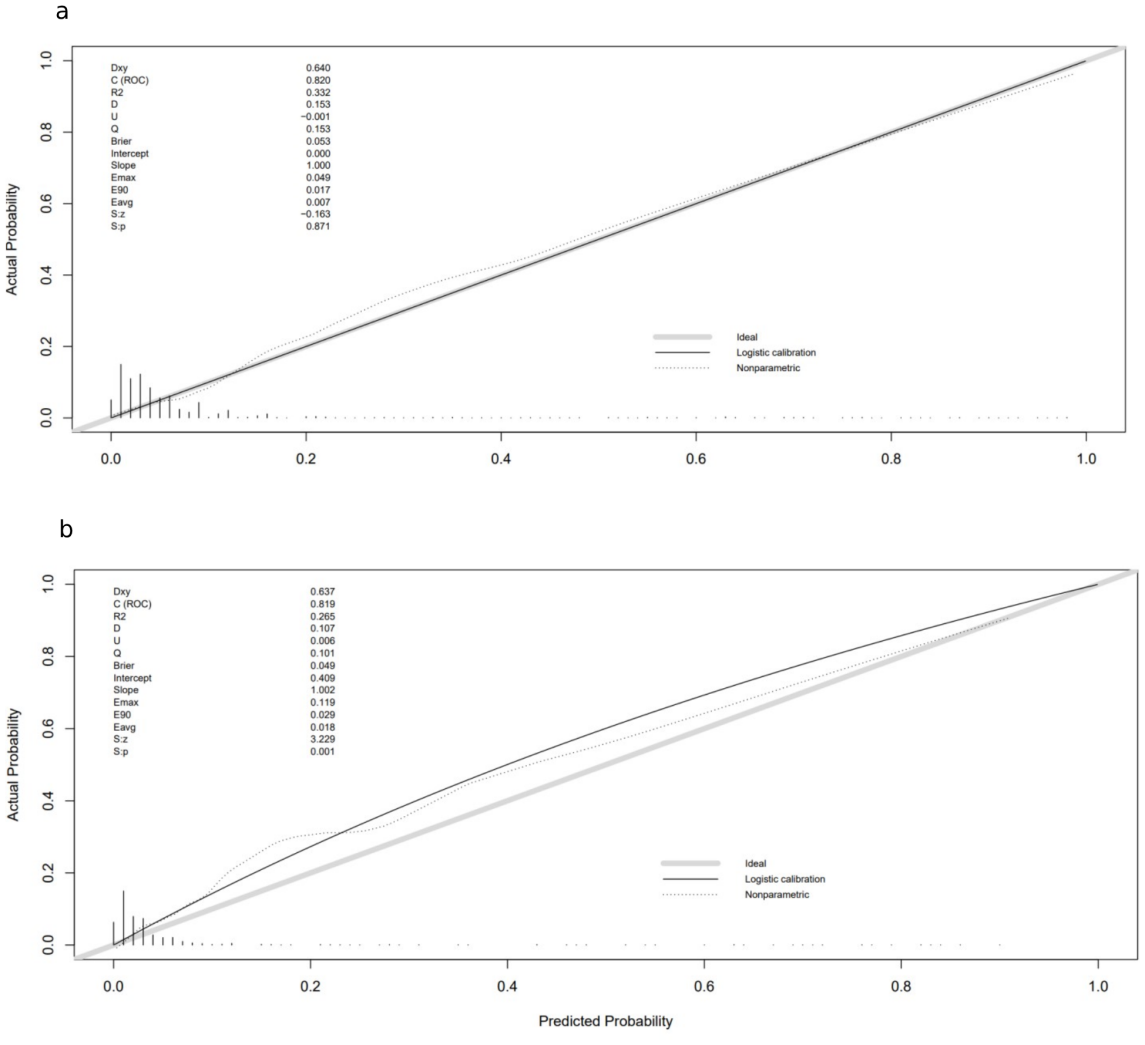
Calibration curves in training and validation sets: **(A)** calibration curve in the training cohort; **(B)** calibration curve in the validation cohort. The gray thick line represents a perfect prediction by an ideal model; the black dashed line indicates the target parameter, and the solid black line shows the performance of the model. Using bootstrap resampling (times = 1,000).

**Figure 5:**
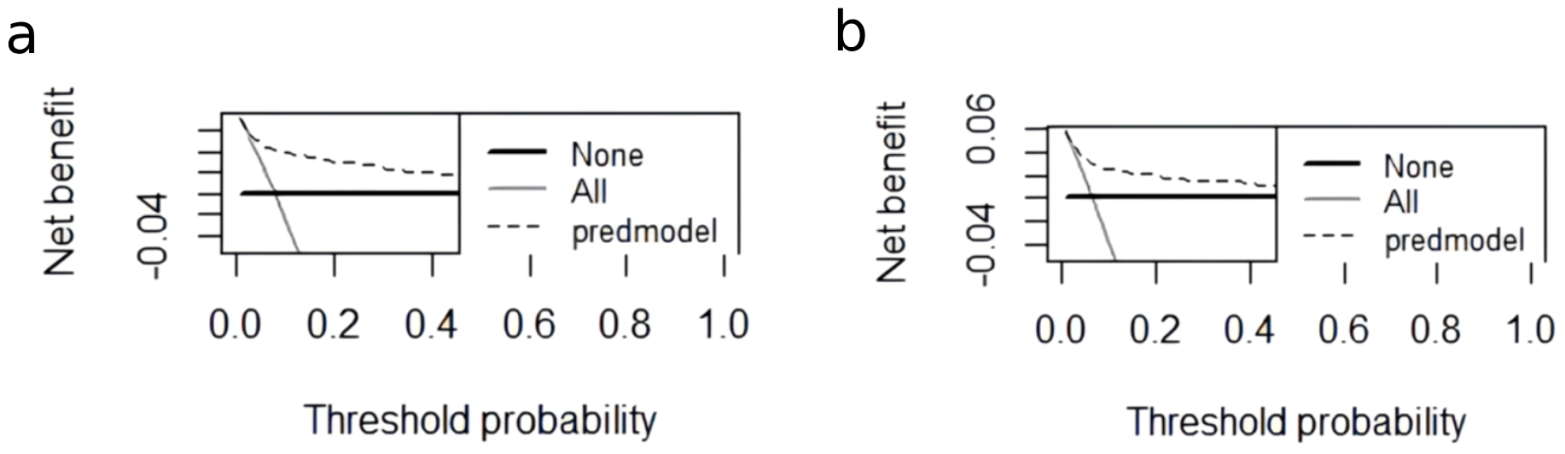
The DCA curve of the nomogram in the training and validation cohorts. **(A)** The decision curve of the nomogram for CRT risk prediction in the training cohort. **(B)** The decision curve of the nomogram for CRT risk prediction in the validation cohort. The black dashed line is the prediction model; the gray solid line shows all samples were intervened, and the black solid line horizontal line shows all samples did not intervene. The graph depicts the expected net benefit of each patient relative to the nomogram in predicting CRT formation risk. With the extension of the model curve, the net benefit increases. Using bootstrap resampling (times = 1,000).

A nomogram for CRT prediction was constructed based on the independent risk factors identified by multivariable logistic regression analysis (**Figure 2A**). **Figure 2B** describes the prediction probability of CRT for the patients. The ROC curve analysis showed a good prediction value of the nomogram in the training (**Figure 3A**, AUC: 0.832 [95% CI: 0.802–0.862]) and validation (**Figure 3B**, AUC: 0.827 [95% CI: 0.783–0.871]) sets. There were no significant differences between the two sets (Delong test: *p* = 0.941). The C-index was 0.856 in the validation process, indicating a good prediction effect. The calibration curves and HL test showed that the nomogram had good calibration performance without over-fitting in the training (**Figure 4A**, HL test *p* = 0.765) and validation (**Figure 4B**, HL test *p* = 0.658) sets. Moreover, the decision curves plotted in the training (**Figure 5A**) and validation (**Figure 5B**) sets showed that the nomogram had clinical benefits. Decision curves calculate the clinical net benefit of a predictive model compared with the default strategies of treating all or no patients. The curves in the present study suggest that, except for a small range of low preferences, the prediction model leads to higher benefits than the alternative strategies of diagnosing all patients or diagnosing no patients.

The patients were divided into the low-, medium-, and high-risk groups based on their predicted incidence. There were significant differences in the actual incidence of CRT among the three risk groups in the training (**Supplementary Figure S2A**) and validation (**Supplementary Figure S2B**) sets (both *p* < 0.001).

## Discussion

In this study, we retrospectively analyzed the characteristics of 4,691 cancer patients to explore the risk factors for CRT and constructed a nomogram for CRT prediction. The results showed that the nomogram had good predictive value and calibration abilities in predicting CRT among cancer patients with a CVC. The findings may help identify patients with a high risk of CRT early.

Some previous studies focused on CRT formation prediction using machine learning methods. Hao et al. Developed a nomogram prediction model for peripherally inserted central catheter (PICC)-related thrombosis prediction and achieved a C-index greater than 0.7 (20). Lin et al. developed a nomogram model for cancer patients with chemotherapy and achieved an AUROC of 0.761 in the validation cohort (21). Nevertheless, those studies were performed with relatively small cohorts (*N* < 1,000) and limited disease states. Their models showed inferior performance compared with the present study, which used large-scale data.

Based on the nomogram model, hypercoagulation was the most important factor in predicting CRT formation. Indeed, a hypercoagulation state is considered an underlying mechanism for thrombosis formation (22). Patients with malignant tumors, particularly those with advanced TNM stages, may experience hypercoagulability due to tumor cells activating the coagulation system (23). Meanwhile, cancer patients often receive long-term CVC infusions, which may cause endothelial damage, further increasing their risk of CRT formation (24). In this study, a history of thrombosis was combined with a past hypercoagulable state in the multivariable linear regression analysis, and the results confirmed that a history of thrombosis/hypercoagulability was an important risk factor for predicting CRT occurrence.

The relationship between the single risk factors identified by the multivariable logistic regression model in this study was analyzed in previous studies (25, 26). Nevertheless, these factors were selected and combined to develop a hybrid nomogram model that could provide accurate prediction performance based on multi-dimensional risk factors. These factors included baseline characteristics such as activity states and TNM stage, biochemical indicators like PLT and D-dimer, pathological states (e.g., hyperlipidemia, hypercoagulability, and co-infection), and external factors (e.g., hormone use and operation history). Risk factors used in the nomogram model exhibited that CRT is a comprehensive pathological condition that requires further study. Six features were included in a previous nomogram-based study, and a C-index of 0.709 (20) was obtained. However, the nine-feature nomogram model developed here achieved a C-index of 0.856.

How hyperlipidemia influences atherosclerosis and thrombosis has been extensively investigated in the past few decades. Atherosclerosis is significantly influenced by hyperlipidemia, which could increase the risk of thrombosis (27). In addition, hyperlipidemia-induced PLT hyperactivity contributes to prothrombosis state development through a CD36-mediated signaling cascade (28). A study suggested that the activation of the coagulation system was associated with the catheter in patients after CVC, which leads to a hypercoagulable state of blood and eventually to the formation of CRT (24). The present study confirmed the close relationship between hyperlipidemia and CRT in patients with tumors who undergo IJV catheterization.

Many cancer patients received hormone therapy as part of their antitumor adjuvant therapy (29, 30). The association between hormone use and hypercoagulability was confirmed previously (31). The present study validated the association of hormone use with increased CRT formation. Therefore, the risk of thrombotic events (CRT, stroke, and myocardial infarction) should be assessed at the patient level with CVC insertion patients before hormone use.

According to the nomogram model, a history of operation provides the lowest contribution to CRT formation prediction. Nevertheless, the results also proved a strong correlation between operation history and CRT formation, in accordance with a recent study, which showed that after chest surgery, 75% of patients developed CRT (32, 33).

This study underwent a two-step feature selection process using univariable and multivariable linear regression models (29, 30). The sample size was calculated with an events per variable (EPV) equal to 5, and the final sample size largely exceeded the expected one. It is well known that large cohorts could improve the performance and robustness of machine learning models (31, 34). Using over 3,000 subjects as the training set, the established model showed great generalization ability and exhibited state-of-the-art performance on CRT formation prediction. The proposed nine-feature nomogram model only included characteristics that were easy to assess in a clinical scenario. No expensive examination is needed to generate an accurate CRT prediction. The innate visualization ability of the nomogram also provides a distinct explanation for different features. That could be useful for clinicians when an explanation is needed.

Although the results were encouraging, this study still had several limitations. First, this study was a single-center retrospective study. A multicenter study is still preferable to evaluate and verify the findings because it yields high-level evidence for clinical application. Second, the selection of the patients in this retrospective study was biased due to the use of inclusion/exclusion criteria, leading to a selection bias. A prospective study would still be needed to validate and confirm the nomogram. Furthermore, all retrospective studies are subject to an information bias since only the data in the charts can be analyzed. Third, due to the objective existence of asymptomatic CRT, the study did not incorporate asymptomatic CRT because it is challenging for clinicians to identify it. If asymptomatic CRT and additional risk factors were included in future studies, the CRT prediction performance may have been even better.

In conclusion, the validated nomograms accurately predict CRT occurrence in cancer patients. To the authors’ knowledge, this was the first study to construct a nomogram for CRT prediction among cancer patients based on such a large-scale dataset. The large dataset used in this study improved the performance and stability of the nomogram model. The findings are expected to help clinicians improve individual treatment plans, manage patients, make clinical decisions, and guide management strategies.

## Data Availability

The original contributions presented in the study are included in the article/**Supplementary Material**. Further inquiries can be directed to the corresponding author.
