# Supplementary material for: Establishment and validation of a predictive nomogram for central venous catheter-related thrombosis in cancer patients: a retrospective nested case-control study
Source: Front Oncol. 2024 Aug 16;14:1418273. doi: 10.3389/fonc.2024.1418273 (PMC11361954; doi:10.3389/fonc.2024.1418273)
Supplement: Supplementary file 1 [file DataSheet1.doc]

**Supplementary Materials**

**Establishment and validation of a predictive nomogram for central venous catheter-related thrombosis in cancer patients: a retrospective nested case-control study**

**Running title: Nomogram for thrombosis prediction**

Xuexing Wang, Xiao Dai, Yuan He, Jie Chu, Yufeng Wang

**
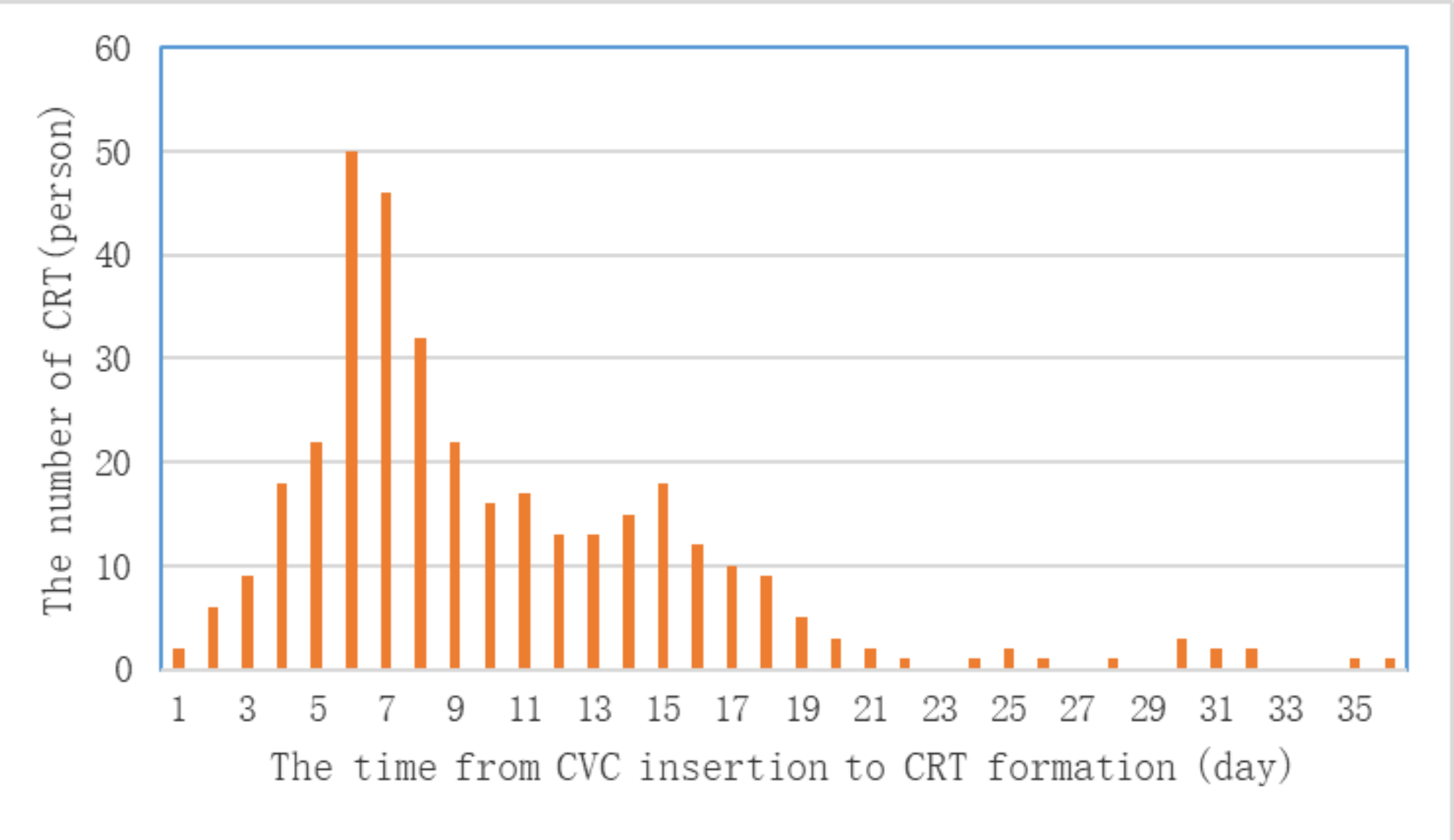
**

**Figure S1. The time distribution between central venous catheter(CVC) insertion to the onset of thrombosis.**

**
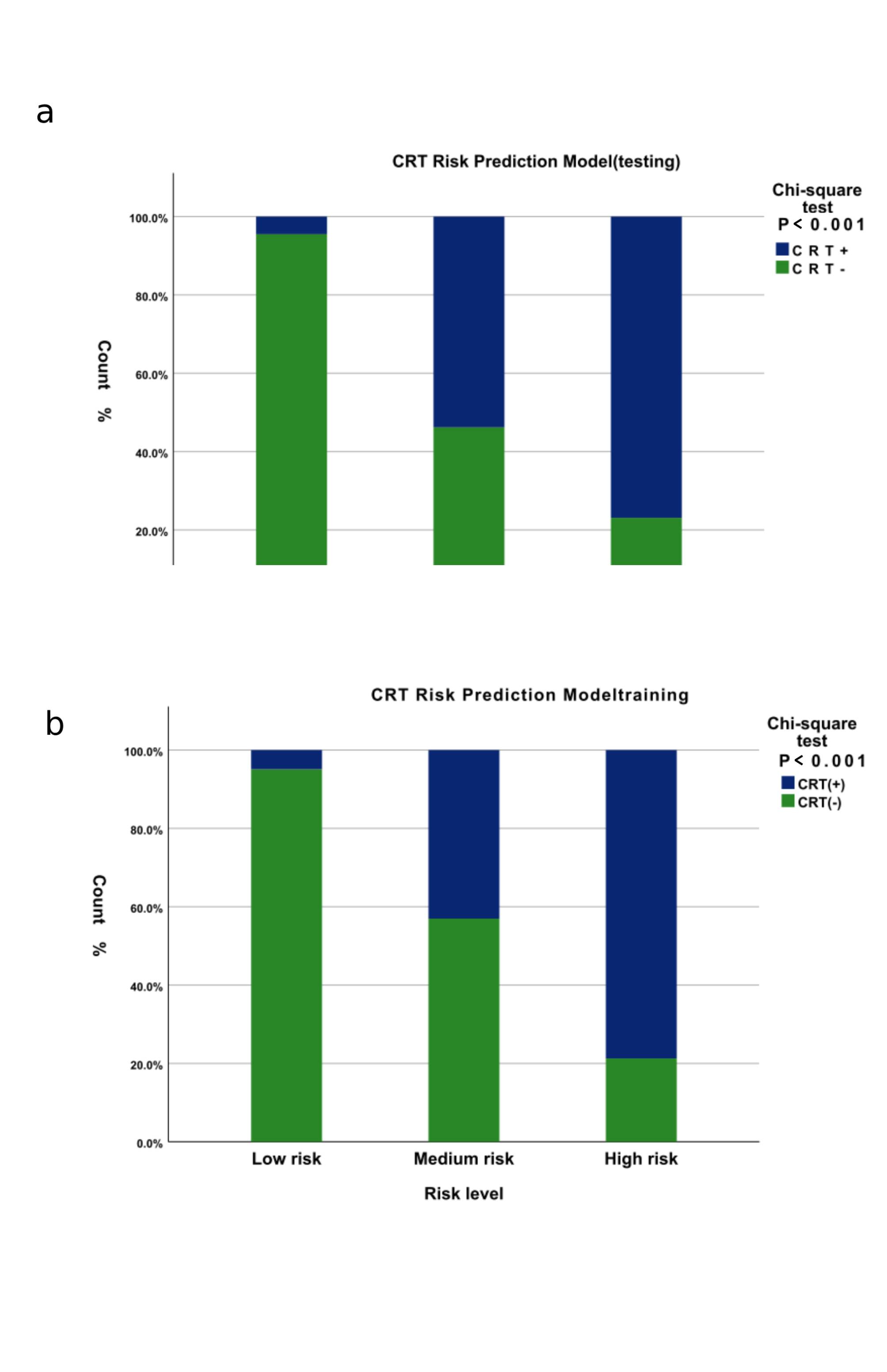
**

**Figure S2. Comparing of actual incidence among patients with different risk for CRT.** a). The training set. b). The Validation set.

**Table S1.** **Variables assignments**

| **Characteristics** | **Variables assignments** | | | | |
| --- | --- | --- | --- | --- | --- |
|  | **0** | **1** | **2** | **3** | **4** |
| **CRT Status** | **Negative** | **Positive** |  |  |  |
| **Gender** |  | **Male** | **Female** |  |  |
| **Age(years)** |  | **≤55** | **>55** |  |  |
| **Number of CVC insertions** |  | **≤2** | **>2** |  |  |
| **Activity amount** | **Hardly** | **Frequently** |  |  |  |
| **History of chemotherapy** | **No** | **Yes** |  |  |  |
| **TNM stage** |  | **I** | **II** | **III** | **IV** |
| **KPS(points)** |  | **>80** | **≤80** |  |  |
| **BMI(kg/㎡)** |  | **≤18.4** | **18.5-23.9** | **24.0-27.9** | **≥28** |
| **Smoking history** | **No** | **Yes** |  |  |  |
| **Drinking history** | **No** | **Yes** |  |  |  |
| **History of blood transfusion** | **No** | **Yes** |  |  |  |
| **Operation history** | **No** | **Yes** |  |  |  |
| **Co-infection** | **No** | **Yes** |  |  |  |
| **Hormone use** | **No** | **Yes** |  |  |  |
| **Hypertension** | **No** | **Yes** |  |  |  |
| **Diabetes mellitus** | **No** | **Yes** |  |  |  |
| **Hyperlipidemia** | **No** | **Yes** |  |  |  |
| **Thrombosis/Hypercoagulability history** | **No** | **Yes** |  |  |  |
| **RBC (10^12^/l)** |  | **<3.5** | **3.5-5.5** | **>5.5** |  |
| **WBC (10^9^/l)** |  | **<4** | **4-10.0** | **>10** |  |
| **PLT (10^9^/l)** |  | **<100** | **100-300** | **>300** |  |
| **HGB (g/l)** |  | **<110** | **≥110** |  |  |
| **PT (s)** |  | **≤12.1** | **>12.1** |  |  |
| **INR** |  | **<0.84** | **0.84-1.06** | **>1.06** |  |
| **APTT (s)** |  | **≤31.3** | **>31.3** |  |  |
| **TT** |  | **<14** | **≥14** |  |  |
| **FIB** |  | **<4** | **≥4** |  |  |
| **ATIII** |  | **≤75** | **>75** |  |  |
| **FDP** |  | **<5** | **5-10** | **>10** |  |
| **D-Dimer(mg/l)** |  | **0-0.55** | **0.56-1.00** | **1.01-2.00** | **2.01~3.00 (3.01-4.00=5, >4.00=6)** |
| **Albumin (g/l)** |  | **>40** | **≤40** |  |  |
| **Albumin/Globulin** |  | **≤1.2** | **>1.2** |  |  |
| **Total bilirubin** |  | **≤17.1** | **>17.1** |  |  |
| **Total cholesterol** |  | **<5.18** | **≥5.18** |  |  |
| **Fasting Blood Glucose** |  | **<6.11** | **≥6.11** |  |  |

**Table S2. Demographic and clinical characteristics among all patients**

| **Characteristic** | **Entire cohort**  **No. (%)** | **Training set (n=3284)**  **No. (%)** | **Validation set (n=1407)**  **No. (%)** | **P** |
| --- | --- | --- | --- | --- |
| **Age (years)** | **53.86±12.04** | **53.79±12.12** | **54.01±11.86** | **0.832** |
| **Gender** |  |  |  | **<0.001** |
| **Male** | **1998(42.6)** | **1140(34.7)** | **858(61)** |  |
| **Female** | **2693(57.4)** | **2144(65.3)** | **549(39)** |  |
| **TNM stage** |  |  |  | **0.011** |
| **I** | **1224(26.1)** | **822(25.0)** | **402(28.6)** |  |
| **II** | **1121(23.9)** | **769(23.4)** | **352(25.0)** |  |
| **III** | **978(20.8)** | **703(21.4)** | **275(19.5)** |  |
| **IV** | **1369(29.2)** | **990(30.1)** | **378(26.9)** |  |
| **Type of cancer** |  |  |  | **0.03** |
| **Genital cancers** | **1597(34.0)** | **1289(39.3)** | **308(21.9)** |  |
| **Respiratory cancers** | **1417(30.2)** | **838(25.5)** | **579(41.2)** |  |
| **Digestive cancers** | **1081(23.0)** | **753(22.9)** | **328(23.3)** |  |
| **Head and neck system tumors** | **212(4.5)** | **158(4.8)** | **54(3.8)** |  |
| **Motior system cancers** | **65(1.4)** | **52(1.6)** | **13(0.9)** |  |
| **Other cancers*** | **319(6.9)** | **194(5.9)** | **125(8.9)** |  |
| **Number of CVC insertions** |  |  |  | **<0.001** |
| **≤2** | **2126(45.3)** | **1348(41)** | **778(55.3)** |  |
| **>2** | **2565(54.7)** | **1936(59)** | **629(44.7)** |  |
| **Activity amount** |  |  |  | **0.052** |
| **Hardly** | **80(1.7)** | **63(1.9)** | **17(1.2)** |  |
| **Frequently** | **4611(98.3)** | **3229(98.1)** | **1390(98.8)** |  |
| **History of chemotherapy** |  |  |  | **<0.001** |
| **No** | **1057(22.5)** | **656(20.0)** | **401(28.5)** |  |
| **Yes** | **3634(77.5)** | **2628(80.0)** | **1006(71.5)** |  |
| **Operation history** |  |  |  | **0.144** |
| **No** | **1865(39.8)** | **1325(40.3)** | **540(38.4)** |  |
| **Yes** | **2825(60.2)** | **1959(59.7)** | **866(61.5)** |  |
| **History of blood transfusion** |  |  |  |  |
| **No** | **4492(95.8)** | **3110(94.7)** | **1382(98.2)** | **<0.001** |
| **Yes** | **199(4.2)** | **174(5.3)** | **25(1.8)** |  |
| **KPS** |  |  |  |  |
| **>80 points** | **807(17.2)** | **619(18.8)** | **188(13.4)** | **<0.001** |
| **≤80 points** | **3884(82.8)** | **2665(81.2)** | **1229(86.6)** |  |
| **BMI(kg/m^2^)** |  |  |  | **<0.001** |
| **<18.5** | **521(11.1)** | **437(13.3)** | **84(6.0)** |  |
| **18.5-23.99** | **2806(59.8)** | **2003(61.0)** | **803(57.1)** |  |
| **24.0-27.99** | **1088(23.2)** | **674(20.5)** | **4014(29.4)** |  |
| **≥28** | **276(5.9)** | **170(5.2)** | **106(7.5)** |  |
| **Smoking history** |  |  |  | **<0.001** |
| **No** | **3410(72.7)** | **2544(77.5)** | **866(61.5)** |  |
| **Yes** | **1281(27.3)** | **740(22.5)** | **541(38.5)** |  |
| **Drinking history** |  |  |  | **<0.001** |
| **No** | **3911(83.4)** | **2819(85.8)** | **1092(77.6)** |  |
| **Yes** | **780(16.6)** | **465(14.2)** | **315(22.4)** |  |
| **Co-infection** |  |  |  |  |
| **No** | **4209(89.7)** | **2914(88.7)** | **1295(92.0)** | **<0.001** |
| **Yes** | **482(10.3)** | **370(11.3)** | **112(8.0)** |  |
| **Hormone use** |  |  |  |  |
| **No** | **2368(50.5)** | **1548(47.1)** | **820(58.3)** |  |
| **Yes** | **2323(49.5)** | **1736(52.9)** | **587(41.7)** |  |
| **Hypertension** |  |  |  | **0.033** |
| **No** | **4030(85.9)** | **2842(86.5)** | **1188(84.4)** |  |
| **Yes** | **661(14.1)** | **442(13.5)** | **219(15.6)** |  |
| **Diabetes mellitus** |  |  |  |  |
| **No** | **4440(94.6)** | **3122(95.1)** | **1318(93.7)** | **0.021** |
| **Yes** | **251(5.4)** | **162(4.9)** | **89(6.3)** |  |
| **Hyperlipidemia** |  |  |  |  |
| **No** | **3998(85.2)** | **2785(84.8)** | **1213(86.2)** | **0.115** |
| **Yes** | **693(14.8)** | **499(15.2)** | **194(13.8)** |  |
| **Thrombosis/Hypercoagulability history** |  |  |  |  |
| **No** | **4501(95.9)** | **3137(95.5)** | **1364(96.9)** | **0.013** |
| **Yes** | **190(4.1)** | **147(4.5)** | **43(3.1)** |  |
| ****lymph system tumors ; urinary system tumors ; endocrine system tumors ; nervous system tumors ; skin tumors*** | | | | |

**Table S3. Univariate logistic regression analysis for CRT in the training set**

| **Characteristics** | **OR(95%CI)** | **P** |
| --- | --- | --- |
| **Age** |  |  |
| **≤55** | **Reference** | |
| **>55** | **1.728(1.340-2.227)** | **<0.001** |
| **TNM** |  |  |
| **I** | **Reference** | |
| **II** | **1.35(0.865-2.105)** | **0.186** |
| **III** | **1.801(1.172-2.767)** | **0.007** |
| **IV** | **3.038(2.078-4.441)** | **<0.001** |
| **Activity amount** |  |  |
| **Hardly** | **Reference** | |
| **Frequently** | **0.053(0.031-0.089)** | **<0.001** |
| **Operation history** |  |  |
| **No** | **Reference** | |
| **Yes** | **1.477(1.128-1.933)** | **0.005** |
| **KPS** |  |  |
| **>80 points** | **Reference** | |
| **≤80 points** | **0.494(0.374-0.653)** | **<0.001** |
| **Co-infection** |  |  |
| **No** | **Reference** | |
| **Yes** | **3.464(2.575-4.695)** | **<0.001** |
| **Hormone use** |  |  |
| **No** | **Reference** | |
| **Yes** | **2.234(1.698-2.939)** | **<0.001** |
| **History of blood transfusion** |  |  |
| **No** | **Reference** | |
| **Yes** | **2.032(1.304-3.167)** | **0.002** |
| **Hypertension** |  |  |
| **No** | **Reference** | |
| **Yes** | **1.762(1.283-2.422)** | **＜0.001** |
| **Diabetes mellitus** |  |  |
| **No** | **Reference** | |
| **Yes** | **1.780(1.105-2.868)** | **0.018** |
| **Hyperlipidemia** |  |  |
| **No** | **Reference** | |
| **Yes** | **2.846(2.149-3.769)** | **＜0.001** |
| **Thrombosis/Hypercoagulability history** |  |  |
| **No** | **Reference** | |
| **Yes** | **27.218(18.883-39.234)** | **＜0.001** |
| **RBC (10^12^/l)** |  |  |
| **<3.5** | **Reference** | |
| **3.5-5.5** | **0.463(0.340-0.630)** | **＜0.001** |
| **>5.5** | **0.159(0.021-1.178)** | **0.072** |
| **WBC (10^9^/l)** |  |  |
| **<4** | **Reference** | |
| **4-10** | **0.936(0.666-1.316)** | **0.704** |
| **>10** | **1.948(1.315-2.885)** | **0.001** |
| **PLT (10^9^/l)** |  |  |
| **<100** | **Reference** | |
| **100-300** | **0.465(0.272-0.794)** | **0.005** |
| **>300** | **0.864(0.498-1.500)** | **0.604** |
| **HGB (g/l)** |  |  |
| **<110** | **Reference** | |
| **≥110** | **0.565(0.433-0.739)** | **<0.001** |
| **PT (s)** |  |  |
| **≤12.1** | **Reference** | |
| **>12.1** | **1.622(1.168-2.253)** | **0.004** |
| **INR** |  |  |
| **<1.0** | **Reference** | |
| **≥1.0** | **1.973(1.527-2.550)** | **<0.001** |
| **FIB** |  |  |
| **<4** | **Reference** | |
| **≥4** | **1.769(1.370-2.285)** | **<0.001** |
| **ATIII** |  |  |
| **≤75** | **Reference** | |
| **>75** | **0.282(0.188-0.422)** | **<0.001** |
| **FDP** |  |  |
| **<5** | **Reference** | |
| **5-10** | **0.887(0.657-1.198)** | **0.887** |
| **>10** | **4.818(3.368-6.891)** | **<0.001** |
| **D-Dimer** |  |  |
| **0-0.55** | **Reference** | |
| **0.56-1.00** | **2.814(1.499-5.280)** | **0.001** |
| **1.01-2.00** | **6.046(3.363-10.870)** | **<0.001** |
| **2.01-3.00** | **1.810(1.055-3.104)** | **0.031** |
| **3.01-4.00** | **11.021(5.512-22.037)** | **<0.001** |
| **>4.00** | **13.039(7.259-23.420)** | **<0.001** |
| **Albumin(g/L)** |  |  |
| **≤40** | **Reference** | |
| **>40** | **0.482(0.371-0.625)** | **<0.001** |
| **Albumin/Globulin** |  |  |
| **≤1.2** | **Reference** | |
| **>1.2** | **0.531(0.384-0.733)** | **<0.001** |
| **Total Bilirubin** |  |  |
| **≤17.1** | **Reference** | |
| **>17.1** | **1.721(1.184-2.502)** | **0.004** |
| **Fasting Blood Glucose** |  |  |
| **<6.11** | **Reference** | |
| **≥6.11** | **1.421(1.025-1.971)** | **0.035** |
